# Structure and regulation of the cellulose degradome in *Clostridium cellulolyticum*

**DOI:** 10.1186/1754-6834-6-73

**Published:** 2013-05-08

**Authors:** Chenggang Xu, Ranran Huang, Lin Teng, Dongmei Wang, Christopher L Hemme, Ilya Borovok, Qiang He, Raphael Lamed, Edward A Bayer, Jizhong Zhou, Jian Xu

**Affiliations:** 1 BioEnergy Genome Center, CAS Key Laboratory of Biofuels and Shandong Key Laboratory of Energy Genetics, Qingdao Institute of Bioenergy and Bioprocess Technology, Chinese Academy of Sciences, Qingdao, Shandong 266101, China; 2 Institute for Environmental Genomics, Department of Botany and Microbiology, University of Oklahoma, Norman, OK 73072, USA; 3 Department of Molecular Microbiology and Biotechnology, Tel Aviv University, Ramat Aviv 69978, Israel; 4 Department of Civil and Environmental Engineering, University of Tennessee, Knoxville, TN 37996, USA; 5 Department of Biological Chemistry, Weizmann Institute of Science, Rehovot 76100, Israel

**Keywords:** Cellulose degradation, Transcription, Two-component systems, Catabolite control proteins, CcpA-like, LacI family

## Abstract

**Background:**

Many bacteria efficiently degrade lignocellulose yet the underpinning genome-wide metabolic and regulatory networks remain elusive. Here we revealed the “cellulose degradome” for the model mesophilic cellulolytic bacterium *Clostridium cellulolyticum* ATCC 35319, via an integrated analysis of its complete genome, its transcriptomes under glucose, xylose, cellobiose, cellulose, xylan or corn stover and its extracellular proteomes under glucose, cellobiose or cellulose.

**Results:**

Proteins for core metabolic functions, environment sensing, gene regulation and polysaccharide metabolism were enriched in the cellulose degradome. Analysis of differentially expressed genes revealed a “core” set of 48 CAZymes required for degrading cellulose-containing substrates as well as an “accessory” set of 76 CAZymes required for specific non-cellulose substrates. Gene co-expression analysis suggested that Carbon Catabolite Repression (CCR) related regulators sense intracellular glycolytic intermediates and control the core CAZymes that mainly include cellulosomal components, whereas 11 sets of Two-Component Systems (TCSs) respond to availability of extracellular soluble sugars and respectively regulate most of the accessory CAZymes and associated transporters. Surprisingly, under glucose alone, the core cellulases were highly expressed at both transcript and protein levels. Furthermore, glucose enhanced cellulolysis in a dose-dependent manner, via inducing cellulase transcription at low concentrations.

**Conclusion:**

A molecular model of cellulose degradome in *C. cellulolyticum* (*Ccel*) was proposed, which revealed the substrate-specificity of CAZymes and the transcriptional regulation of core cellulases by CCR where the glucose acts as a CCR inhibitor instead of a trigger. These features represent a distinct environment-sensing strategy for competing while collaborating for cellulose utilization, which can be exploited for process and genetic engineering of microbial cellulolysis.

## Background

Lignocellulosic biomass is the most abundant biopolymers on earth, yet recalcitrance to hydrolysis has hampered its exploitation for renewable bioenergy and biomaterials [1,2]. In nature, direct hydrolysis of lignocellulose is carried out exclusively by microorganisms. Cellulolytic clostridia, which are ubiquitous in cellulosic anaerobic environments, represent a major paradigm for efficient biological degradation of cellulosic biomass [3,4]. Many of these anaerobes digest cellulose via a cell surface-attached extracellular enzymatic complex called the cellulosome where primarily catalytic components (including glycoside hydrolases, carbohydrate esterases and polysaccharide lyases) are integrated onto a non-catalytic macromolecular scaffoldin subunit [5,6]. These host cells [4,7,8] and their cellulolytic machineries [9] are being exploited in the production of cellulosic biofuels by a variety of approaches, notably consolidated bioprocessing (CBP; [10]). However, the structure and regulation of the “cellulose degradome”, i.e., the genome-wide metabolic and regulatory networks underpinning cellulose degradation, remain poorly understood. Identifying genetic components of the degradome and elucidating how their activities are organized and regulated *in vivo* should form the basis for developing natural or engineered cellulases and their host cells for efficient production of cellulose-based biofuels.

*Clostridium cellulolyticum*, a Gram-positive cellulosome-producing anaerobe of the Family 4 (or Cluster III) of Clostridia [11], has become a model organism for the study of mesophilic cellulolysis [12-14]. In addition to cellulose, it grows on a wide variety of carbohydrates including soluble cellodextrins, glucose, xylan, xylose, arabinose, fructose, galactose, mannose and ribose [15-17]. By sequencing its complete genome and comparing its transcriptomes and extracellular proteomes collected under different growth conditions (cellulose and its derivative mono- and di-saccharides), we report here a genome-wide, single-nucleotide resolution bacterial cellulose degradome for the *C. cellulolyticum* strain H10 or ATCC 35319 (abbreviated here as *Ccel*). Two functional tiers (core and accessory) of CAZymes were revealed that are respectively transcriptionally regulated by a Carbon Catabolite Repression (CCR) mechanism and two-component systems (TCSs). Surprisingly, instead of suppressing cellulase transcription, glucose promotes cellulolysis by inducing cellulase transcription at low concentrations while by promoting cell growth at high concentrations. A molecular model of the cellulose degradome in *Ccel* was proposed which revealed the substrate-specificity of CAZymes and the transcriptional regulation of core cellulases by CCR where the glucose acts as a CCR inhibitor instead of a trigger. These features represent a distinct environment-sensing strategy for competing while collaborating for cellulose utilization, which can be exploited for process and genetic engineering of cellulolysis.

## Results

### Genomic features of a mesophilic cellulose degrader

The complete genome of *Ccel* consists of a single circular 4,068,724 bp chromosome with a GC content of 37.4%. It encodes 3390 proteins, 63 tRNAs and 24 rRNAs (Additional file 1: Table S1; GenBank Accession Number NC_011898; [18]). CAZymes are the critical enzymes that cleave, build and rearrange oligo- and polysaccharides [19]. Relative to other mesophilic cellulosome-producing clostridia such as *C. acetobutylicum*[20] and *C. cellulovorans*[21], *Ccel* harbors the least number of CAZyme genes (149 genes), but features the largest portfolio of cellulosomal genes which consists of 62 dockerin-encoding genes and three cohesin-encoding genes (*cipC*/Ccel_0728, *orfX*/Ccel_0733 and Ccel_1543). The cellulosomal enzymes in *Ccel* are diverse and complementary in functions, which included cellulases, hemicellulases (xylanases, mannanases, and arabinofuranosidases), pectate lyases and chitinases [22]. Moreover, the cellulosomal genes in *Ccel* tend to physically cluster along the chromosome, representing an organizational pattern distinct from *C. thermocellum* ATCC 27405 [23]. Among the 65 cellulosomal genes in total, we identified several clusters: i) the “*cip-cel*” gene cluster (Ccel_0728-0740) that encodes the major cellulosome components (including scaffoldin), most of which encode cellulases [24], ii) a second cluster of 14 genes (Ccel_1229-1242) encoding exclusively secreted dockerin-containing proteins, which are probably involved in hemicellulose degradation and herein named the “*xyl-doc*” gene cluster [22], iii) three small clusters (each with two genes) encoding cellulosomal enzymes (Man26A/Ccel_0752-Cel9P/Ccel_0753, PL10/Ccel_1245-CE8/Ccel_1246 and Ccel_1655-1656), and iv) one cluster (Ccel_1549-1550) of one non-cellulosomal and one cellulosomal genes.

### Structure of the cellulose degradome in *C. cellulolyticum*

To identify the components of the cellulose degradation in *Ccel*, we started by characterizing the populations of transcripts in *Ccel* cultures under a variety of carbon sources using RNA-Seq. The carbohydrate substrates tested included i) cellulose and its derivatives glucose and cellobiose, ii) hemicellulose (using xylan from oat spelts as a representative substrate) and its derivative xylose, and iii) corn stover, a natural plant-derived residue which consists of both cellulose and hemicellulose (Additional file 2: Figure S1A). In total, 12.4 million reads were uniquely mapped to the genome, representing combined sequence coverage of 223X. After removing rRNA reads, for each of the substrates tested, 74.3% to 84.2% of the reads were mapped to previously annotated coding regions, and the remaining were either upstream of a coding sequence (CDS; thus putatively identifying a 5′-untranslated region (5′-UTR)) or mapped to unannotated or potentially mis-annotated regions.

In total, a large majority (86.0%) of the genome was actively transcribed under at least one of the conditions, while 59.5%, 59.8%, 69.3%, 67.1%, 36.4% and 63.2% of the genome were transcribed under glucose, cellobiose, xylose, cellulose, xylan and corn stover, respectively. Furthermore, 8521 regions of a total of 1.16 Mb (28.5% of the genome) were expressed under each of the substrates tested, representing a “core transcriptional glycobiome”. These regions exhibited a scattered pattern along the genome. On the other hand, 167 regions (142 overlapping with CDS and 25 within intergenic regions) with a total of merely 14,338 bp (only 0.34% of the genome) were expressed under only one substrate (129 regions were found to be cellulose-specific, among which 18 were intergenic). Thus, specificity of the transcribed loci in response to carbon substrates was manifested in the relative level of transcription, instead of their presence or absence.

For each CDS, its Normalized Transcript Abundance (NTA) under a particular substrate was determined (Additional file 3: Table S2) and then compared across the various carbon substrates supporting *Ccel* cultivation (Additional file 2: Figure S1B). We defined the “cellulose degradome” as the collection of genes transcribed (NTA > 1) under cellulose. The “cellulose-specific degradome” was defined as those required for degradation of cellulose but not for that of cellulose derivatives (glucose and cellobiose); specifically, a gene was included only when i) its NTA under cellulose is greater than 1, and ii) the ratio of NTA between cellulose and glucose and that between cellulose and cellobiose are both greater than 2 and the *p* values (statistical significance of differential expression) are both lower than 0.001.

Those CDS encoding core metabolic functions (macromolecule biosynthetic process, protein biosynthesis and primary metabolic process) are enriched in the cellulose degradome of *Ccel* as compared to the complete proteome encoded in the genome. Moreover, except for nucleic acid binding (GO:0003676), various Gene Ontology (GO) categories related to environmental sensing, gene regulation and polysaccharide metabolism are also enriched in the cellulose degradome of *Ccel.*

**Figure 1:**
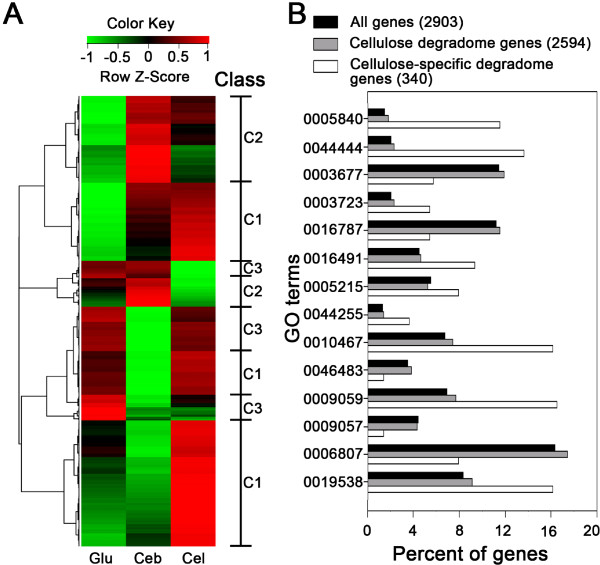
**Structure of cellulose degradome in *C. cellulolyticum.*** (**A**) Hierarchical clustering analysis of 650 genes that exhibit substrate-specific gene expression under glucose (Glu), cellobiose (Ceb), and cellulose (Cel). A Row Z-Score measures the relationship between the NTA of a gene under a given condition and the mean NTA of the gene under the multiple conditions compared (i.e. the row). A Row Z-score of 0 means the NTA is equal to the mean NTA. Positive (or negative) Row Z-score indicates the degree to which the NTA is higher (or lower) than the mean. The corresponding classes (C1, C2 or C3) were indicated. (**B**) Functional profiles of the genes that exhibit substrate-specific gene expression. Those GO terms specifically enriched or depleted in the cellulose-specific degradome were shown (0005840: ribosome; 0044444: cytoplasmic part; 0003677: DNA binding; 0003723: RNA binding; 0016787: hydrolase activity; 0016491: oxidoreductase activity; 0005215: transporter activity; 0044255: cellular lipid metabolic process; 0010467: gene expression; 0046483: heterocycle metabolic process; 0009059: macromolecule biosynthetic process; 0009057: macromolecule catabolic process; 0006807: nitrogen compound metabolic process; 0019538: protein metabolic process).

Differentially Expressed Genes (DEGs; including both positively and negatively regulated) among substrates were further identified. At the threshold of *P* < 0.001, 1043 DEGs were identified from the 15 pair-wise comparisons of the six substrates. Most DEGs were involved in energy production and conversion, carbohydrate transport and metabolism, and translation. In total, 650 genes were differentially expressed between any two of the conditions of glucose (monosaccharide), cellobiose (disaccharide) and cellulose (polysaccharide), which formulated three main groups (via hierarchical clustering; Figure 1A; Additional file 4: Table S3). The first class (Class C1; 342 genes) showed the highest NTA under cellulose. Of them, 63 genes showed high NTA (Z-score > 0) in glucose. In comparison with cellulose degradome genes, the remaining 279 genes in the cellulose-specific class (cellulose-specific “degradome”) showed enrichment for ribosomal proteins (GO:0005840), oxidoreductase activity (GO:0016491), RNA binding (GO:0003723), gene expression (GO:0010467), macromolecule biosynthetic processes (GO:0009059) and protein metabolic processes (GO:0019538), etc (Figure 1B). The second class (Class C2) included 207 genes showing the highest NTA under cellobiose. Within this class are 17 genes showing high NTA under cellulose and 25 under glucose. The remaining 165 genes were enriched with ion transport (GO:0006811), protein binding (GO:0005515) and nucleotide metabolic processes (GO:0006139). A third class of 101 genes (Class C3) showed the highest NTA under glucose among the carbon sources, where catabolic processes (GO:0009056), carbohydrate metabolic processes (GO:0005975) and carbohydrate binding (GO:0030246) were enriched.

Surprisingly, 145 of the 148 CAZymes (except Ccel_0750, Ccel_0920 and Ccel_2109) encoded by *Ccel* genome were not found in the cellulose-specific degradome due to their similar transcriptional levels under cellulose and glucose, suggesting an unusual link between monosaccharide catabolism and cellulose degradome in this organism. To further probe the links among the substrate-specific degradomes, we performed co-expression analysis of all CAZyme genes encoded in *Ccel* genome under the different substrates.

### Regulation of the cellulose degradome in *C. cellulolyticum*

**Figure 2:**
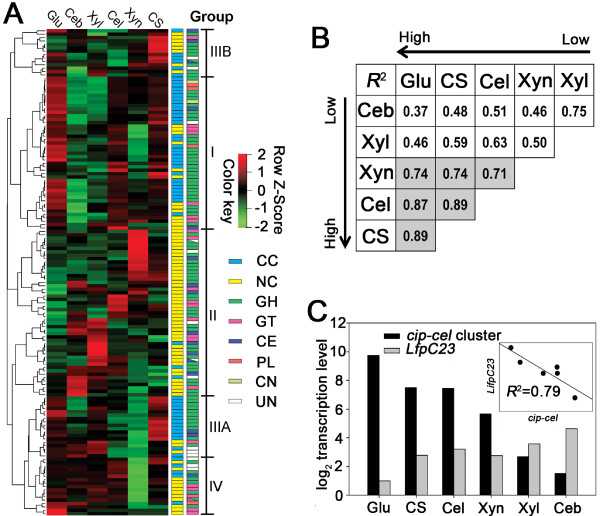
**CCR-regulation of cellulose degradome components in *C. cellulolyticum*.** (**A**) Expression profiles of CAZymes under the selected carbon sources (glucose (Glu), cellobiose (Ceb), xylose (Xyl), cellulose (Cel), xylan (Xyn) and corn stover (CS)) were clustered into four groups by hierarchical clustering analysis and gene function. The structural (cellulosomal component (CC) and noncellulosomal enzyme (NC)) and functional (glycoside hydrolase (GH), glycosyltransferase (GT), carbohydrate esterase (CE), polysaccharide lyase (PL), cellulosomal noncatalytic subunit (CN) and unknown function enzyme (UF)) characteristics of CAZmes were distinguished by different color-blocks. (**B**) The expression correlation of the 50 cellulosomal genes under various carbon sources. Correlation coefficients (*R*^2^) were calculated (*n* = 50) for all cellulosomal genes except the *“xyl-doc”* cluster. The arrows indicated the order in average transcript abundance (from low to high) of the genes under the different conditions. (**C**) Correlation of NTA between “*cip-cel*” cluster and *lfpC2-IfpC3* (Ccel_2999 -3000) under different carbon sources.

Based on their substrate-dependent transcription patterns, the 143 CAZyme genes (except Ccel_0428, Ccel_0429, Ccel_2073, Ccel_2123 and Ccel_2442 which were not expressed under any of the carbon sources and a cohesin-encoding gene Ccel_1543) were clustered into four different groups (Figure 2A; Additional file 5: Table S4).

### Carbon catabolite repression (CCR)

Group I includes 45 genes that showed higher expression levels under glucose, cellulose, xylan and corn stover relative to cellobiose and xylose, which included the “*cip-cel*” gene cluster (Ccel_0728-0740). Genes of this group mainly encode cellulosomal components, including scaffoldin subunits and major enzymatic subunits, which belong to GH families 5, 9, 26 and 48 and others involved in cellulose degradation. Surprisingly, most of the cellulosomal genes except the “*xyl-doc*” cluster belong to this group. Interestingly, the NTAs of all the 50 cellulosomal genes (not including the “*xyl-doc*” cluster) were correlated to each other, with highest correlation coefficients (*R*^2^ > 0.7) under glucose, cellulose, xylan and corn stover (Figure 2B; in grey).

Transcription of Group I CAZymes appears to be regulated by the carbon catabolite repression (CCR), as suggested its synchronic yet distinct differential patterns among substrates that featured a negative correlation between NTAs and growth rate. For example, the order in average NTA of Group I genes was cellulose (or corn stover or glucose) > xylan > xylose > cellobiose (Figure 2A), while that in growth rate was cellobiose > xylose > xylan > cellulose (or corn stover or glucose) (Additional file 2: Figure S1A). Catabolite control protein A (CcpA) is thought to be one of the key CCR regulators in *Bacillus subtilis*[25]. CcpA belongs to the LacI family of transcriptional regulators and binds selectively to specific DNA sequences (referred to as catabolite-responsive element, or *cre*) [26,27]. Recently a 18-nt *cre*-like motif with 3 mismatches (TGTGTACGCGTTTATATT) was found upstream of the “*cip-cel*” gene cluster in *Ccel*; it was shown to be involved in regulating at least *cipC* by a CCR mechanism [28]. The *Ccel* genome has five genes (Ccel_1005, Ccel_1438, Ccel_2999, Ccel_3000 and Ccel_3464) that encode putative regulators of the LacI-family. In *Ccel*, the protein sequence of Ccel_1005 has the highest identity and similarity (34% and 55%, respectively) to that of *B. subtilis* CcpA. Four other proteins are slightly less similar (e.g., 25% and 46% in identity and similarity for Ccel_2999; 26% and 44% for Ccel_3000) to CcpA but more conserved in DNA-binding helix-turn-helix (HTH) domains. We therefore propose to use CcpA for Ccel_1005, while the other four LacI-family regulators are named herein as LfpC1, LfpC2, LfpC3 and LfpC4 (**L**acI **f**amily **p**roteins in ***C***. *cellulolyticum*). Surprisingly, the expression levels of two neighboring genes, *lfpC2* and *lfpC3* (Ccel_2999 and Ccel_3000, respectively) were strongly negatively correlated with average expression levels of the “*cip-cel*” gene cluster with different carbon sources, and related coefficient (*R*^2^) reaches 0.79 (Figure 2C). Meanwhile, certain *cre* consensus-like sequences, possibly recognized by CcpA, LfpC1, LfpC2, LfpC3 and LfpC4, were determined via MEME [29] based on predicted DNA-binding motifs of these transcription factors [30]; the two center positions of the predicted putative 16-nt motifs were limited to “CG” owing to the conservation of this nucleotide pair in the CcpA binding site consensus sequences (Additional file 6: Figure S2). Genome-wide scanning of intergenic regions using FIMO [29] revealed 110 putative *cre* sites (18, 17, 20, 27 and 28 sites recognized respectively by CcpA, LfpC1, LfpC2, LfpC3 and LfpC4) in *Ccel* (Additional file 7: Table S5). However, only seven CAZyme genes on their upstream regions included a *cre* site motif, which was recognized by LfpCs but not by CcpA. Five of the seven genes (*cel9Q*/Ccel_0231, *cipC*/Ccel_0728, Ccel_0755, Ccel_1207 and Ccel_1439) belong to Group I (Additional file 7: Table S5). Notably, the putative *cre* site (AAGTTAT**CG**TTAATTA) we identified for the “*cip-cel*” cluster was distinct and 87 bp upstream of the previously reported *cre* site [28], suggesting the presence of multiple *cre* sites within the upstream region of the “*cip-cel*” cluster. Thus the majority of cellulosomal genes might be regulated by CcpA-independent CCR, such as GlyR3 [31], CcpC [32] or CcpN [33].

### Two-component systems (TCSs)

Group II includes 49 genes that showed high expression specifically on one substrate (e.g. cellulose, cellobiose, xylose or xylan) (Figure 2A). These genes encode noncellulosomal enzymes from GH10, 51, 94 and other GH and GT families (Additional file 5: Table S4). In particular, the genes encoding xylanases (GH8: Ccel_1258; GH10: Ccel_2319, 2320, 0153), a xylosidase (GH3: Ccel_1139) and arabinofuranosidases (GH51: Ccel_1255 and 1221) were highly expressed specifically under xylan, whereas cellobiose/cellodextrin phosphorylase genes (GH94: Ccel_3412 and 2109) are expressed specifically under cellulose, while hemicellulase genes (GH18: Ccel_2893, 0643 and 2820; GH23: 0815) and some glycosyltransferase genes (Ccel_0486, 3410, 1334, 0333) are expressed specifically under xylose.

Group III is mainly the “*xyl-doc*” gene cluster (Ccel_1229-1242) that exhibited higher expression levels under corn stover than other carbon sources (Figure 2A; Additional file 5: Table S4). The low expression of “*xyl-doc*” cluster genes on xylan from oat spelts indicates that they hydrolyze hemicellulose other than the xylan from oat spelts. They also encode cellulosomal components, which belong to GH43, 27, 10 and other families involved in hemicellulose degradation. The remaining CAZymes are collectively assigned to Group IV, which are mainly non-GH family enzymes, such as members of the GT1 family (Figure 2A; Additional file 5: Table S4).

**Figure 3:**
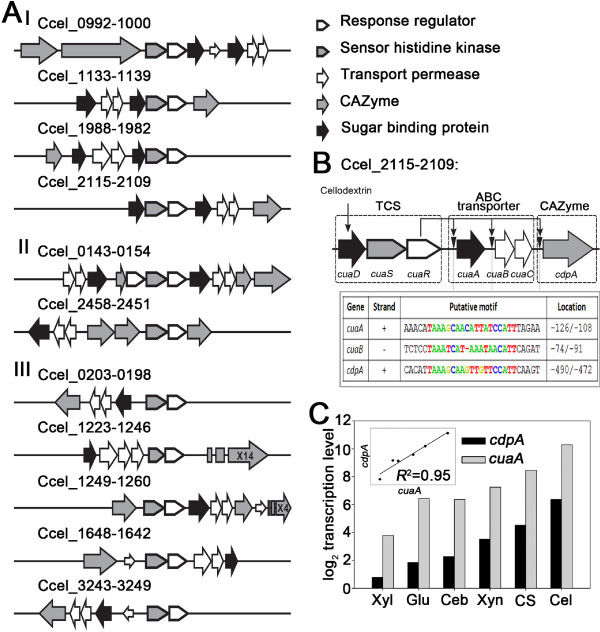
**TCS-regulation of cellulose degradome components in *C. cellulolyticum.*** (**A**) The eleven genomic loci encoding TCSs, ABC transporters and CAZymes. The loci can be classified into three categories, where the TCS operon harbors *sbp2* (Category I), CAZymes (Category II) or no other genes (Category III) as an accessory to the TCS. (**B**) Detailed schematic of Ccel_2109-2115, one of the eleven genomic loci. The regulation of *cuaA*, *cuaBC* and *cdpA* by CuaR was indicated by arrows. The putative 19-nucleotide cuaR-binding motifs upstream of *cua*A, *cua*B and *cdp*A were shown as a table, with their positions relative to the first base of the translation start of the corresponding genes indicated. (**C**) Correlation of transcript levels between *cuaA* and *cdpA* of the Ccel_2109-2115 cluster under various carbon sources.

Further analysis revealed that transcription of 76 CAZymes that include noncellulosomal enzymes (Group II; 49 genes) and cellulosomal hemicellulase components (including “*xyl-doc*” gene cluster; Group III; 27 genes) might be regulated by a TCS mechanism. *Ccel* possesses 37 putative TCSs, eleven of which were flanked by genes encoding Group II and Group III CAZymes and putative sugar ABC transporters (Figure 3A). In these loci, the CAZyme genes exhibited similar expression patterns to ABC transporter genes (if both were highly expressed) under the carbon sources tested. Thus CAZymes of Group II, Group III and ABC transporters appeared to be co-regulated by TCSs. Our results were confirmed by a recent study which showed that one TCS (XydS/XydR; Ccel_1227/1228) transcriptionally regulates Group III CAZymes (the “*xyl-doc*” gene cluster; [34]). Meanwhile, genes encoding sugar-binding proteins (SBP) were found in two loci (named *sbp1* and *sbp2*, respectively) that encoded ABC transporter genes and TCS genes (Figure 3A; TCS-loci Category I). For example, Ccel_2109-2115 encoded one CAZyme (Ccel_2109; encoding a cellodextrin phosphorylase named *cdpA*), **c**ellulose-**u**tilization **a**ssociated ABC transporters (Ccel_2112-2110, named *cuaA*, *cuaB* and *cuaC*), and TCS (Ccel_2115-2113, named *cuaD*, *cuaS* and *cuaR*) (Figure 3B). Expression of the *cuaA* (Ccel_2112), encoding a potentially periplasmic high-affinity solute-binding protein, exhibited substrate-specificity (with the highest level under cellulose) and is strongly correlated with that of the *cdpA* under different carbon sources (*R*^2^ = 0.97) (Figure 3C). However, the *sbp2* gene (*cuaD*) was expressed constitutively with TCS as an operon at a low level (<0.2% of *sbp1* (*cuaA*) on cellulose) under each of the carbon sources. Moreover, upstream of *cdpA*, *cuaA* and *cuaB*, there is a conserved sequence motif that might serve as a putative binding site of CuaR (a TCS response regulator harboring an “AraC”-family DNA-binding domain) (Figure 3B). Therefore, SBP2 may be a “signal collector” of TCS. When an extracellular sugar molecule is specifically bound to SBP2, the complex formed may activate the sensor histidine kinase, which can phosphorylate a cognate response regulator (e.g. CuaR). Subsequently the phosphorylated regulator may promote the expression of genes encoding Group II of CAZymes and ABC transporters which specifically hydrolyze polysaccharides and transport the hydrolysates.

Thus via CCR control, cellulosomal genes (except the “*xyl-doc*” cluster) were induced under recalcitrant carbon sources (cellulose and corn stover) and repressed under cellobiose and xylose. On the other hand, via TCS regulation, noncellulosomal enzymes, cellulosomal hemicellulases encoded by the “*xyl-doc*” cluster and ABC transporters were induced in a substrate-specific manner.

Therefore, the CAZyme components of the cellulose degradome can be classified into two categories: i) the “core” proteins (Group I) which are required for cellulose degradation, and ii) the “accessory” proteins (Group II and III) which are not required for cellulose degradation. Furthermore, transcriptional regulation of the core is associated with CCR, while that of the accessory is linked to TCS.

### Activation of cellulose degradation by glucose in *C. cellulolyticum*

Curiously, the NTA of most of the Group I genes were over four times higher under glucose than under cellobiose, xylose or xylan (Figure 2A, Additional file 3: Table S2), suggesting glucose induced transcriptionally at least part of the cellulose degradome. To test whether the NTA upregulation led to elevation in protein abundance, the secreted proteomes of *Ccel* under glucose and cellobiose were analyzed via label-free quantitative proteomics using LC-MS/MS. At the protein level, the number and yield of cellulosomal components under glucose were significantly higher than under cellobiose: for example, 13 cellulosomal components were identified under glucose, but only five components were found under cellobiose (Additional file 8: Table S6).

**Figure 4:**
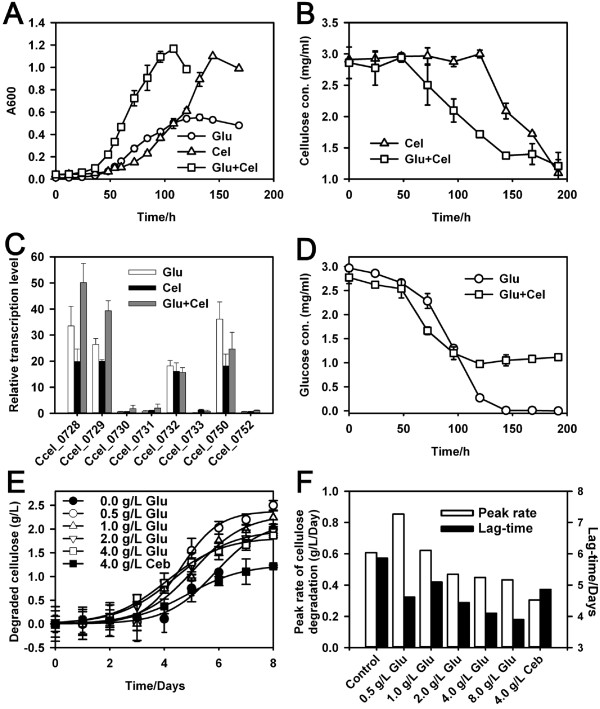
**Activation of cellulose degradation by glucose in C. cellulolyticum.** The bacterium was cultured on a mixture of 3 g/L glucose (Glu) and 3g/L cellulose (Cel), 3 g/L singular carbon source of Glu, or 3 g/L singular carbon source of Cel. The growth curves (**A**) and concentrations of residual cellulose (**B**) and glucose (**D**) in broth were measured. The transcriptional level of several cellulosomal genes (including those in the cel-cip cluster) was quantified using qRT-PCR (**C**). To test the dependence of the cellulolytic activity on glucose concentration, the bacterium was cultured on cellulose mixed with series concentrations (0.5, 1.0, 2.0, 4.0 and 8.0 g/L) of glucose; two conditions (singular cellulose and cellulose mixed with 4.0 g/L cellobiose) were used as control. The data point of 8g/L glucose was not shown. Amount of degraded cellulose was measured, and the curves of cellulose degradation were fitted by sigmoidal equation using data from three biological replicates (**E**). The peak cellulose degradation rate and the lag-time (the number of days it took to reach the peak rate) under each culture condition were both shown (**F**). Means of three biological replicates were shown.

To test the hypothesis of glucose-based promotion of cellulase expression and cellulose degradation, we cultured *Ccel* on singular or mixed carbon source of cellulose (3 g/L) and glucose (3g/L). Under dual-substrate, arrival of mid-log phase was ~24 hours earlier than that under glucose alone and ~48 hours earlier than that under cellulose alone (Figure 4A), suggesting faster cellulose degradation when glucose is present. Moreover cellulose degradation under dual-substrate was ~50 hours faster than that under cellulose alone (Figure 4B), while glucose utilization rate under dual-substrate was similar to that under glucose alone (Figure 4D). Quantitative RT-PCR (qRT-PCR) revealed that the transcription level of the eight genes (six of them were from the *cip-cel* cluster) encoding major cellulosomal components in Group I under glucose or glucose-cellulose was significantly higher than (for six genes) or equal to (for two genes) that under cellulose (Figure 4C). In particular, the two main cellulosomal genes in the *cip-cel* cluster, Ccel_0728 (*cipC*) and Ccel_0729 (*cel48F*), were transcribed at significantly higher level (2-fold) under dual-substrate than under cellulose-alone. Thus glucose enhanced degradation of cellulose by inducing expression of the cellulosomal genes in *Ccel*.

To test whether the inductive effect of glucose on cellulose degradation is dependent on glucose concentration, we cultured *Ccel* on cellulose which was mixed with a gradient of glucose (0.5-8.0 g/L) or cellobiose (4g/L). The culture under cellulose-alone was used as control (Figure 4E). The peak cellulolysis rate decreased under incremental concentrations of the glucose supplement (Figure 4F): the rates under lower glucose-supplements (0.854 and 0.622 g/L/Day under 0.5 and 1.0 g/L respectively) were up to 41% higher than that of cellulose-alone (0.607 g/L/Day), while those under higher glucose-supplements (0.469, 0.449 and 0.434 g/L/Day under 2, 4 and 8 g/L respectively) were 23 ~ 29% lower than that of control (but still higher than that under 4 g/L cellobiose (0.305 g/L/Day)). On the other hand, the lag-time (the time taken to reach the peak cellulose degradation rate) under higher glucose-supplements (4.44, 4.10 and 3.90 Day under 2, 4 and 8 g/L respectively) was faster by1.42-1.96 Day than that of control (5.86 Day), while that under lower glucose (0.5 and 1 g/L) was only 0.76-1.24 Day faster than that of control. Thus glucose supplementation promotes cellulose degradation by inducing cellulase transcription at low concentrations.

Such glucose induction of cellulase transcription and cellulolysis and its dependency on glucose concentration appeared to be quite unique as they have not been previously reported in this and any other microorganisms [28,35]. Several lines of evidence suggested glucose as an edible but not preferred carbon source of *Ccel*, which potentially explains the surprising trait: i) *Ccel* growth was much slower under glucose than under cellobiose [36] or xylose and xylan (Additional file 2: Figure S1A); ii) Under glucose-cellulose mixture *Ccel* cells did not exhaust glucose, which remained at ~1 g/L from mid- to late-log phase (Figure 4D); iii) The NTA of putative glucokinase genes (Ccel_0700 and Ccel_3221, the first enzyme in the Embden-Meyerhof pathway) under glucose were 36 ~ 58% lower than under other soluble sugars such as xylose and cellobiose (Additional file 3: Table S2); iv) Under higher glucose-supplements (4 and 8 g/L), the peak cellulolysis rates (0.449 and 0.434 g/L/Day) were higher than that under 4 g/L cellobiose-supplement (0.305 g/L/Day; Figure 4F), consistent with the report that repression of the *cip-cel* cluster by cellobiose was more drastic than by glucose [28]. Therefore, the activation of cellulase transcription by a non-preferred carbon source (i.e., glucose) and inhibition by a preferred substrate (i.e., cellobiose) in *Ccel* can be explained by the CCR mechanism.

### A molecular model of the cellulose degradome in *C. cellulolyticum*

**Figure 5:**
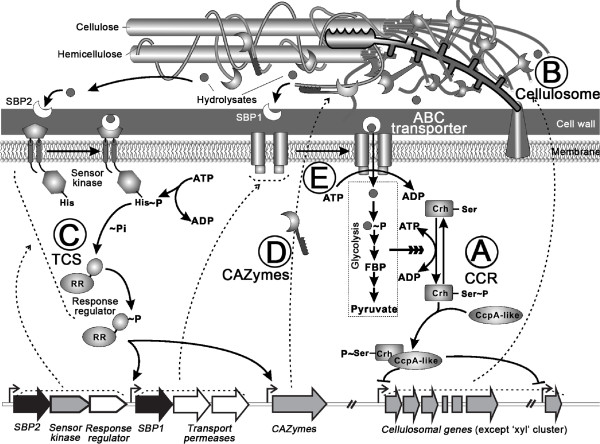
**Cellular model of cellulose degradome in *Clostridium cellulolyticum*.** The cell employs CCR (**A**) to sense intracellular level of glycolytic intermediates and controls the core cellulose-degrading machinery that mainly includes cellulosomal components (**B**). On the other hand, TCS systems (**C**) sense extracellular soluble sugars and respectively regulate carbon-substrate specific CAZymes (**D**) and transporters (**E**). Due to the synergy of cellulosomal enzymes and the substrate-specific CAZymes, cellulose is degraded efficiently and the resulted soluble di- and mono-saccharides transported into cell.

In view of the above, we propose a structural and regulatory model for the cellulose degradome in *Ccel* (Figure 5). In this model, utilization of cellulose requires at least three functional classes of proteins, including CAZymes that catalyze cellulose hydrolysis, ABC transporters of the hydrolysates and the signal transduction systems (CCR and TCS). The cellular degradation of cellulose consists of five steps: (*A*) When *Ccel* is grown on mineral medium with a lignocellulose substrate (including both pentose and hexose) or non-preferred monosaccharides (e.g., glucose) as the sole carbon source, the CCR mechanism is relieved, leading to low levels of intracellular glycolytic intermediates. Consequently, a homologue of the phosphocarrier proteins (Crh (catabolite repression HPr)-like protein, Ccel_0806) remains dephosphorylated and prevents the CcpA homologues, such as LfpC*2* (Ccel_2999) or LfpC3 (Ccel_3000), from inhibiting the transcription of the major cellulosomal genes (except the “*xyl-doc*” gene cluster) or activates their expression via other regulators. (*B*) As a result, the cellulosomal components are expressed, secreted and assembled into cellulosomes anchored on the cell surface, which catalyzes hydrolysis of the lignocellulose. (*C*) The soluble saccharides resulted from lignocellulose hydrolysis are captured by sugar-binding proteins (SBP2); the signal is transduced into cells via the intramembrane-sensing histidine kinase of the TCSs. The histidine kinase phosphorylates the response regulator, which activates expression of ABC transporters and CAZyme genes. (*D*) The temporal synergy and functional complementarity between the transcriptionally upregulated CAZymes may then accelerate lignocellulose degradation generating the release of soluble sugars. (*E*) ABC transporters, whose transcription is also activated via the TCS, transport and feed the extracellular soluble sugars into the glycolysis pathway. The resultant high concentrations of glycolytic intermediates would inhibit the expression of cellulosomal genes via CCR, thus closing this five-step cycle of regulated cellulose degradation.

## Discussion

Efficient conversion of lignocellulosic biomass to transportation biofuels such as ethanol is a leading candidate solution among alternatives to fossil fuels because of its sustainability and rural economic benefits [37]. To maximize the energy and cost efficiency in the conversion process, schemes such as consolidated bioprocessing (CBP) were proposed, where hydrolysis of lignocellulosic biomass, co-utilization of pentose and hexose, and robust ethanol fermentation are built into a single bioreactor [10]. Cellulolytic clostridia are among the leading CBP candidates due to their wide carbon substrate range that include cellulose. They produce a wide variety of CAZymes with different specificities for lignocellulose hydrolysis, yet most of which remain functionally uncharacterized. Here we demonstrated their functional classification into the ‘core’ and ‘accessory’, which aimed respectively at the major constituent-crystalline cellulose and other variable constituents of lignocellulosic biomass. The observed differential NTAs between core and accessory enzymes and within each of the two classes might underlie the stoichiometry of the protein products. Thus the results can potentially serve as a blueprint for construction of potent cellulase systems (*in vitro* or *in vivo*) tuned or optimized for the targeted substrate by matching the abundance of core enzymes, the type and abundance of accessory enzymes as well as their stoichiometry.

Furthermore, we have untangled a collaborative regulatory network involving CCR and TCS that regulate the ‘core’ and ‘accessory’ respectively. Previous studies observed substrate-dependent differential expression of a few cellulases in *C. cellulovorans*[38] and *C. thermocellum*[39-42], and identified one *cis*-acting element (*cre*) tentatively involved in CCR-based regulatory mechanism in *C. cellulolyticum*[28]. However, the global regulation of cellulolysis remains unknown and the contribution of CCR unclear. This first genome-wide model for cellulose degradation here revealed a functional web of CCR. *First*, 45 CAZymes were found regulated by CCR, suggesting a global regulatory role of CcpA-like proteins. However, as *Ccel* does not seem to encode an HPr ortholog and any ‘cognate’ PTS enzyme EII [28], alternative regulatory systems could be involved in the CCR mechanism in this bacterium. *Second*, there are only five *cre* sites in promoter regions of Group I genes, suggesting that in addition to CcpA-dependent CCR, there are CcpA-independent CCRs involved in regulating Group I genes. *Third*, within the *cip-cel* cluster, few such *cre*-like sites were found, yet the transcriptome profile indicated multiple transcriptional start-sites or post-transcriptional processing sites were present (consistent with previous transcriptional analysis of the *cip-cel* gene cluster [43]), suggesting additional mechanisms controlling the differential transcription of cellulase genes encoded within the cluster.

In natural environments, cellulose which consists of only glucose is a shared component of all types of plant biomass, yet distinct types of hemicelluloses (e.g. xylan, glucuronoxylan, arabinoxylan, glucomannan or xyloglucan) which consists of many different monosaccharides are found in different plants or plant tissues. As cellulose hydrolysis is a shared activity for consuming various plant biomasses, it is efficient to employ CCR which responds to intracellular level of glycolytic intermediates to modulate cellulase transcription. On the contrary, expression of hemicellulases is only needed for certain types of plant biomass, thus TCS which senses the presence of extracellular sugars was adopted for transcriptional activation of the hemicellulase genes. Therefore, in *Ccel*, the CCR-mediated monitoring of cellular needs for energy and the TCS-mediated sensing of environmental substrate-availability likely ensure both sensitivity to environmental nutrients and the efficiency of cellulose degradome.

Surprisingly, contrary to most known CCR models such as those found in *Escherichia coli* and *B. subtilis* and many pathogenic bacteria (where glucose serves as a CCR trigger; [44]), in *Ccel* the glucose instead acts as a CCR inhibitor, where the presence of glucose relieves the inhibitory effect of CCR, consequentially resulting in transcriptional activation of Group I CAZymes for cellulolysis. Our results are inconsistent with an earlier report [28] that glucose activated CCR and inhibited expression of the *cip-cel* cluster, which was likely due to some differences in the specific *Ccel* laboratory clones of strain H10 tested. Furthermore, we showed that the inductive effect of glucose on cellulase transcription and cellulose degradation is dependent on glucose concentration, where glucose promotes cellulose degradation by inducing cellulase transcription at low doses while by promoting cell growth at higher doses.

These traits appear to be quite novel. Among the mesophilic phylogenetic relatives of *Ccel*, *C. acetobutylicum*[20,45] and *C. cellulovorans* both prefer glucose; in the latter cellulases was transcriptionally repressed under glucose but was derepressed upon glucose exhaustion [38]. In *C. thermocellum*, cellobiose is the preferred carbon source as in *Ccel*[4], yet its cellulase transcription is probably activated by alternative σ factors released by their cognate anti-σ factors that might sense availability of extracellular cellulose [46]. In fungi, cellobiose serves as the inducer of cellulase expression in *Trichoderma reesei*[47] and *Asperillus* species [48], cellotriose or cellotetraose in *Phanerochaete chrysosporium*[49] and cellodextrins in *Neurospora crassa*[50].

These distinct traits might convey to *Ccel* advantages in its natural niche, where cellulose is abundant, glucose scarce and competition for edible sugars keen. *First*, it avoids direct competition of cellulolytic organisms (who are often in minority, e.g., in the rumen only ~10% of the bacteria are cellulolytic [51]) with non-cellulolytic bacteria for carbon source. For most heterotrophic bacteria studied to-date, glucose is the preferred (or primary) carbon source [52]. The varied diet preference might lead to a more sustainable ecosystem [53]. *Second*, the uptake of cellobiose or cellodextrins into the cell is more energy-efficient than glucose: the former requires less ATP per glucose residue, and the breakdown of cellodextrins into glucose-1-phosphate by the intracellular cellodextrin phosphorylase ,conserves ATP [54]. *Third*, as glucose is soluble, induction of cellulases upon low concentration of glucose might allow *Ccel* to detect nearby cellulolytic activities and thus respond rapidly to cellulose availability.

This trait might find applications in CBP [54] where microbes act singularly or collaboratively to convert lignocellulosic biomass to fuel molecules such as ethanol. As many non-cellulolytic yet fuel-fermenting organisms (e.g., *E.coli*, *Zymomonas mobile* and *Saccharomyces cerevisiae*) prefer glucose, the complementary diet of *Ccel* would make it a suitable CBP partner. This trait can also be exploited to improve cellulase production and cellulolysis in *Ccel*.

Intricate structure and precise control of the cellulose degradome such as those found in *Ccel* here are likely the norm rather than an exception in nature (cellulolytic organisms span a wide phylogenetic and ecological spectrum [55]), yet the degree of conservation and the evolutionary links among them remain unknown. For example, in the related *C. thermocellum*, a distinct mechanism involving multiple alternative σ^I^-like factors [46] was found modulating transcription of cellulosomal genes, suggesting a surprising degree of divergence for cellulolysis regulation in cellulolytic clostridia. Comparing the cellulose degradomes in this and related organisms should help the design and construction of cellular systems for robust and green conversion of lignocellulose to valuable products.

## Conclusions

A molecular model of cellulose degradome in *Ccel* was proposed that revealed the substrate-specificity of CAZymes and their regulatory modes. CCR-related regulators sense intracellular glycolytic intermediates and control the core CAZymes that mainly include cellulosomal components. On the other hand, 11 sets of Two-Component Systems (TCSs) respond to availability of extracellular soluble sugars and respectively regulate most of the accessory CAZymes and associated transporters. Surprisingly, glucose acts as a CCR inhibitor instead of a trigger. Under glucose alone, the core cellulases were highly expressed at both transcript and protein levels. Furthermore, glucose enhanced cellulolysis in a dose-dependent manner, via inducing cellulase transcription at low concentrations. These features represent a distinct environment-sensing strategy for competing while collaborating for cellulose utilization, which can be exploited for process and genetic engineering of microbial cellulolysis.

## Methods

### Strains and culture conditions

*Clostridium cellulolyticum* ATCC 35319 or H10 (*Ccel*) was cultured anaerobically at 35°C in 250 mL flasks with 100 mL working volume of modified DCB-1 medium [56] supplemented with 2.0 g/L of glucose, xylose, cellobiose, or 5.0 g/L of cellulose (Avicel PH101), xylan (from oat spelts) or milled corn stover. A 1% (v/v) inoculum of culture pre-adapted on various substrates in vials was used for inoculation. Cellular growth on glucose, xylose, cellobiose and cellulose was monitored by optical density of the culture at 600 nm (OD_600_), while that on xylan and corn stover was measured based on increase of cellular proteins in the culture, as suspension of substrates interfered with OD_600_ measurement. After lysing cells in NaOH/SDS solution, cell debris were pelleted and removed, then protein concentration in the supernatant was estimated using the BCA assay. Concentrations of residual glucose and cellulose were measured respectively by Megazyme D-glucose kit and phenol-sulfuric acid method as described previously [9].

### Preparation and sequencing of transcriptomes

Total RNA was isolated from cultures harvested at the mid-log phase using RNeasy Mini Kit (Qiagen). Genomic DNA was removed by RNase-Free DNase Set (Qiagen). RNA quality was determined using Bioanalyser (Agilent) and quantified using ND-2000 (NanoDrop Technologies). Message RNA were purified by removing 16S and 23S rRNA from total RNA using MicrobExpress™ Bacterial mRNA Purification kit (Ambion), with the exception that no more than 5 μg total RNA was treated per enrichment reaction. Reduction of 16S and 23S rRNA was confirmed by 2100 Bioanalzyer (Agilent) and gel electrophoresis prior to preparation of cDNA fragment libraries. RNA was reversely transcribed using random primers and Superscript III (Invitrogen) to generate cDNA.

Sequencing libraries for GA-IIx (Illumina, USA) were constructed by shearing the enriched cDNA by nebulization (35psi, 6 min) followed by end-repair with Klenow polymerase, T4 DNA polymerase and T4 polynucleotide kinase (to blunt-end the DNA fragments). A single 39 adenosine moiety was added to the cDNA using Klenow exo and dATP. The Illumina adapters (containing primer sites for sequencing and flowcell surface annealing) were ligated onto the repaired ends of cDNA and gel-electrophoresis was used to separate library DNA fragments from unligated adapters by extraction of the 200–250 bp cDNA fragments. Fragmentation followed by gel electrophoresis was used to separate library DNA fragments and size fragments were recovered using gel extraction at room temperature to ensure representation of AT rich sequences. Libraries were amplified by PCR (18 cycles) with Phusion polymerase. Sequencing libraries were denatured with sodium hydroxide and diluted to 3.5 pM hybridization buffer before loading into a lane of an Illumina GA flowcell. Cluster formation, primer hybridization and single-end, 36 cycle sequencing were performed (Illumina, USA). The efficacy of each stage during library construction was ascertained by quality control which involved measuring the adapter-cDNA on an Agilent DNA 1000 chip. A final dilution of 2 nM of the library was loaded onto the sequencer.

### Transcriptomic analysis

#### Mapping reads to the genome

A customized computational pipeline was developed. Low quality bases located at the end of each read were removed, then the reads were mapped to the *Ccel* genome (GenBank: NC_011898) using SOAP. Reads that did not align uniquely to the genome or were mapped to rRNA genes were discarded. The mismatch number parameter (−v) used in SOAP was 2.

#### Core and accessory transcriptional glycobiome

The “core transcriptional glycobiome” were defined as regions expressed under all of the substrates tested. The “accessory transcriptional glycobiome” were regions expressed under only one carbon substrates. For the latter, two additional criteria were used to filter out potential false positives: (i) not overlapping with other transcribed regions and (ii) average sequencing depth being greater than two.

#### Normalized transcript abundance (NTA)

Transcript abundance (TA) was determined as follows: for each particular gene *j* in the NCBI annotation, the number of unique *k* hits associated with each base in each gene was quantified, overall k values summed which correspond to each base located in gene *j*, and then divided by the length of gene *j* to represent TA of gene *j*. This value was then normalized using each sample’s average sequencing depth (ASD). The normalized TA (NTA) was calculated as: NTA_j_ = TA_j_ / ASD.

#### Estimation of differential expression

Based on each gene’s NTA, an R package (DEGseq) was employed to identify those differential expression genes. The MA-plot-based method was used with random sampling model. The sets of genes were selected for further analysis after the following filters: (1) NTA log_2_ ratios were considered significant when ≧2.0 or ≦ −2.0; (2) positive NTA log_2_ ratios that had numerators below 0.01 were ignored; (3) negative NTA log_2_ ratios that had denominators below 0.01 were not selected; (4) the *P*-value for differential expression was set to be ≦ 0.05.

In addition to those based on intensity ratio and average intensity (MARS), two other methods were employed to evaluate differential expression: Fisher’s exact test and Likelihood ratio test. All these methods were implemented in DEGexp [57]. The overall differential expression calls were highly similar among the methods and in all subsequent analysis, thus differential expression genes validated by these three methods were used for the following analysis.

#### Validation of mRNA-Seq based transcript quantification

To examine the biological reproducibility of RNA-Seq, one pairs of differential cDNA libraries (C2 and C3) were constructed and sequenced as biological replicates of the original cellulose (C1) library. Correlation analysis was performed using Spearman’s rank correction test. The RNA-Seq data was found to be highly and significantly correlated among the three biological replicates (Additional file 9: Figure S3A and B). For evaluating the technical reproducibility, two replicates for the each biological replicate of cellulose samples were sequenced on GA-IIx, which demonstrated reproducibility (Additional file 9: Figure S3C, D, E).

To further validate the mRNA-Seq based transcript quantification, we correlated the results from RNA-Seq (the average NTA for a transcript) with the absolute transcript copy number measured via qRT-PCR. The qRT-PCR was performed using the SYBR Green I on LightCycler®480II using FastStart Universal SYBR Green Master (Roche). Genes selected for this test included Ccel_0270, 0271, 0297, 0298, 0445, 0446, 0597, 0598, 0728, 0729, 0730, 0731, 0732, 0885, 1060, 1608, 1986, 1987, 2065, 2066, 2111, 2112, 1223 and 2485 under two conditions (growing on glucose and cellulose), which encode the subunits of cellulosome and components of ABC transporters. The primer sets for qRT-PCR were listed in Additional file 10: Table S7. Data of qRT-PCR from these studies were normalized against expression of Ccel_0312 which encodes the beta-subunit of DNA-directed RNA polymerase. Relative RNA-Seq read coverage under each condition was normalized against data obtained under glucose. Based on transcript levels of the 24 genes, log-transformed average NTA and Log-transformed qRT-PCR relative transcriptional level were respectively correlated (Additional file 9: Figure S3F, *P* < 0.0001, *R*^2^ = 0.82), indicating that RNA-Seq provides reliable quantitative estimate of NTA.

### Functional comparisons of transcripts and transcriptomes

Association between genes and COG functional groups was based on NCBI (http://www.ncbi.nlm.nih.gov/sutils/coxik.cgi?gi=23673). Non-expressed genes and those not assigned by COG were excluded for further analysis. The COG functional groups with less than 20 expressed genes were discarded for lacking of statistical power. Gene Ontology (GO) categories and InterPro ID were assigned using InterProScan (version 4.7). The number of genes in the *Ccel* genome assigned to each GO term, or its parents in the hierarchy, was totaled. To control difference in the specificity of gene prediction, genes that could not be assigned to a GO category were excluded from the reference sets. The results were compiled and statistical comparisons were made among the numbers of genes assigned to each GO term in different samples.

Putative CAZymes encoded in the genome were identified by comparing each protein model with a library of modules derived from all the entries present in CAZy [19] (http://www.cazy.org/). This library includes catalytic modules involved in hydrolysis, modification or creation of glycosidic bonds (from the enzymes classes GH, CE, PL, and GT), as well as CBMs, dockerin and cohesin modules. Genes harboring these modules were compared based on their transcriptional level.

### Transcription factor-specific operator motif analysis

Sequence homologs of putative CcpA-like regulator (Ccel_1005) and four other LacI-family proteins in *Ccel* (Ccel_1438, Ccel_2999, Ccel_3000 and Ccel_3464) were found from the NR database using the BLASTP server at NCBI and the HTH domains of the above-mentioned *Ccel* proteins as queries (Additional file 6: Figure S2A). The upstream regions of the genes harboring each of the groups of homologous domains were searched for putative operator sites via MEME [29]. The LacI-family transcriptional factors (TFs) were known to form functional dimmers thus the binding sequence motifs of these TFs are 10 ~ 16 bp palindromes [30,58]. A position-specific scoring matrix was created using WebLogo (http://weblogo.berkeley.edu/) by applying MEME to the defined operator regions (Additional file 6: Figure S2B). The matrices were used as input for an automated motif search at a database of upstream sequences of all *Ccel* genes using FIMO (*P* < 0.0001) [29].

### Proteome analysis

Cultures grown on the glucose and cellobiose for proteomics experiments were harvested at the end of the exponential growth stage. The cultures were centrifuged (12,000 g, 4°C, 30 min), and the supernatants filtered through a 0.22-m PES membrane to obtain a cell-free fraction. The cell-free supernatants were concentrated using an ultrafiltration device containing a noncellulosic PES membrane with a 5 kDa molecular weight cutoff (Millipore). Concentrated samples were then pooled, precipitated with 1/4 volume of 100% (w/w) trichloroacetic acid (TCA) and incubated for 60 min at 37°C in 1% SDS, 0.2 M NaOH, and 10 mM DTT. Cysteines were alkylated with 30 mM iodoacetamide at room temperature in the dark for 60 min. Proteins were again precipitated using TCA and resuspended in 50 mM Tris–HCl (pH 7.6), 1M urea and digested overnight at 37°C with sequencing grade trypsin (Promega) with a 50:1 substrate to enzyme ratio. Peptide solutions were acidified with trifluoroacetic acid (TFA) to a final concentration of 0.5% and <50 μg of peptides. Peptides were desalted using C18 reversed-phase extraction using Pierce C-18 spin columns (Thermo) and analyzed by microcapillary LC-MS/MS using a hybrid quadrupole/atmospheric pressure ionization orthogonal accelerated time-of-flight mass spectrometer (MSI-Q-TOF; Bruker Daltonics).

The MS/MS spectra acquired were assigned to specific peptide sequences using Mascot (http://www.matrixscience.com/search_intro.html) with a FASTA proteome database specific to *C. cellulolyticum*. The database contained common contaminant protein entries as well as reversed decoy sequences for assessment of protein-level false discovery rates. Absolute protein abundance within each treatment was estimated from MS/MS spectral counts using Trans-Proteomic Pipeline (TPP) [59].

## Abbreviations

ABC: ATP-binding cassette; ASD: Average sequencing depth; CAZymes: Carbohydrate-active enzymes; CBM: Carbohydrate-binding module; CBP: Consolidated bioprocessing; Ccel: Clostridium cellulolyticum; CcpA: Catabolite control protein A; CCR: Carbon catabolite repression; CDS: Coding sequence; CE: Carbohydrate esterase; Ceb: Cellobiose; Cel: Cellulose; cre: Catabolite-responsive element; CS: Corn stover; DEGs: Differentially expressed genes; DTT: Dithiothreitol; GH: Glycoside hydrolase; Glu: Glucose; GT: Glycosyltransferase; GO: Gene ontology; HTH: Helix-turn-helix; LfpC: LacI family proteins in *C. cellulolyticum*; NTA: Normalized transcript abundance; PL: Polysaccaride lyase; SBP: Sugar-binding protein; TA: Transcript abundance; TCA: Trichloroacetic acid; TCS: Two-component system; TFA: Trifluoroacetic acid; OD600: Optical density at 600 nm; PES: Polyethersulfone; 5′-UTR: 5′-Untranslated region; Xyl: Xylose; Xyn: Xylan.

## Competing interests

The authors declare no conflicts of interest.

## Authors’ contributions

JX, CX, RH, JZ, QH designed research; CX, RH, LT, DW performed research; CX, RH, JX, EAB, RL, IB, CLH analyzed data; CX, RH, JX wrote the paper. All authors read and approved the final manuscript.

## Supplementary Material

Additional file 1: Table S1 General features of the complete genome of *Clostridium cellulolyticum* ATCC 35319 (H10). Click here for file

Additional file 2: Figure S1 Growth curves and transcriptomic overview of *Clostridium cellulolyticum*. (*A*) Growth curves of *Clostridium cellulolyticum* on glucose, xylose, cellobiose, cellulose, xylan and corn stover. Cell growth was monitored by measuring OD_600_ under glucose (Glu, open square), xylose (Xyl, open triangle), cellobiose (Ceb, open circle) and cellulose (Cel, filled circle) and by determining the amount of cellular protein produced on xylan (Xyn, filled triangle) and corn stover (CS, filled square) as described in **Methods**. The symbols indicate the means of three experiments, and the error bars indicate the standard deviations. (*B*) Overview of the *Clostridium cellulolyticum* transcriptome generated by RNA-Seq. Data were normalized by the number of CDS for each function encoded within the entire genome. A ratio of 1 represents transcription of functional class on par with its genome content. A ratio of more than one represents a transcriptionally enriched class, and less than one, depleted. Click here for file

Additional file 3: Table S2 The normalized transcript abundance (NTA) of all genes under various carbon subtrates in *Clostridium cellulolyticum*. Click here for file

Additional file 4: Table S3 *Clostridium cellulolyticum* genes that were differentially expressed between any two of the conditions of glucose, cellobiose and cellulose. Click here for file

Additional file 5: Table S4 The four groups of CAZymes and their relative transcript abundance under the various carbon substrates in *Clostridium cellulolyticum*. Click here for file

Additional file 6: Figure S2 Phylogeny and putative operator motifs of CcpA-like regulators in *C. cellulolyticum*. (*A*) Phylogenetic (Neighbor-joining) tree of the HTH DNA-binding domains of Ccel_1005, Ccel_1438, Ccel_2999, Ccel_3000 and Ccel_3464, and their homologues in other Gram-positive bacteria (**Methods**). Bootstrap values are indicated. (*B*) Putative operator motifs of the *Ccel* LacI-family regulators. Upstream regions of the genes flanking the query and subject genes were inspected for sharing similarity to the *cre* consensus sequence and used to create the Ccel_1005, Ccel_1438, Ccel_2999, Ccel_3000 and Ccel_3464 specific operator motifs. Click here for file

Additional file 7: Table S5 Putative *cre* sites present in the promoter regions of certain *Clostridium cellulolyticum* genes. Click here for file

Additional file 8: Table S6 Label-free quantitation of proteins in cell-free supernatant of *Clostridium cellulolyticum* based on the normalized spectra counts of LC-MS/MS. Click here for file

Additional file 9: Figure S3 Validation of mRNA-Seq based transcript quantification for genome-wide expression profiling. (*A* and *B*) Biological replicates. In the correlation plots, each point indicates the TA of an individual CDS in two biological replicates for cellulose sample. (*C*, *D*, and *E*) Technical replicates. In the correlation plots, each point indicates the TA of an individual CDS in pairwise technical replicates for cellulose sample. (*F*) Real-time quantitative RT-PCR (qPCR) validation of mRNA-Seq based transcript quantification. The induction levels were compared among 24 genes in *C. cellulolyticum*. All genes were randomly selected. The comparison was plotted on log_2_R, which was determined by qRT-PCR (*x* axis) and RNA-Seq (*y* axis). Click here for file

Additional file 10: Table S7 A complete list of PCR primer sets used in this study. Click here for file
