# Supplementary material for: Structure and regulation of the cellulose degradome in Clostridium cellulolyticum
Source: Biotechnol Biofuels. 2013 May 8;6:73. doi: 10.1186/1754-6834-6-73 (PMC3656788; doi:10.1186/1754-6834-6-73)
Supplement: Additional file 1: Table S1 — General features of the complete genome of Clostridium cellulolyticum ATCC 35319 (H10). [file 1754-6834-6-73-S1.doc]

**Table S1. General features of the complete genome of *Clostridium cellulolyticum* ATCC 35319 (H10)**

| **Genome size (base pairs)** | 4,068,725 |
| --- | --- |
| **G+C content (%)** | 37.4 |
| **Open reading frames** | 3578 |
| **Predicted protein encoding sequences** | 3390 |
| **Predicted sequences encoding RNA genes** | 88 |
| **Coding density (%)** | 90.9 |
| **Average gene size (base pairs)** | 1034 |
| **rRNA** | 24 |
| **tRNA** | 63 |
| **CRISPRs loci** | 3^a^ |
| **Small non-coding RNAs** | More than 45^b^ |
| **Pseudo genes** | 100 |
| **Insertion sequence (IS) elements** | 86^c^ |
| **Genes with function prediction (%)** | 2596 (72.6%) |
| **Number of genes in COG (%)** | 2721 (76%) |
| **Number of genes in Pfam (%)** | 2655 (74.3%) |
| **Genes encoding signal peptides (%)** | 705 (19.7%) |
| **Genes encoding transmembrane proteins (%)** | 970 (27.1%) |
| **Genes with orthologs (%)** | 3257 (91.1%) |
| **Genes with paralogs (%)** | 664 (18.6%) |

^a^http://crispr.u-psud.fr/crispr/

^b^Chen et al. (2011) Small RNAs in the genus *Clostridium*. mBio 2: e00340-10.

^c^There are also certain IS remnants that are not in the above number.
