# Supplementary figures and images for: Structure and regulation of the cellulose degradome in Clostridium cellulolyticum
Source: Biotechnol Biofuels. 2013 May 8;6:73. doi: 10.1186/1754-6834-6-73 (PMC3656788; doi:10.1186/1754-6834-6-73)

**A**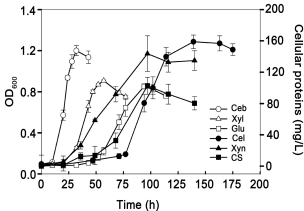**B**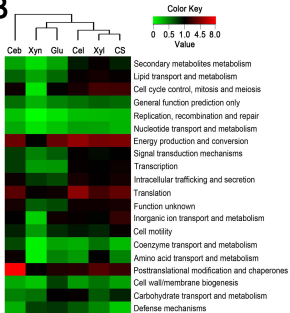

Supplement: Additional file 2: Figure S1 — Growth curves and transcriptomic overview of Clostridium cellulolyticum. (A) Growth curves of Clostridium cellulolyticum on glucose, xylose, cellobiose, cellulose, xylan and corn stover. Cell growth was monitored by measuring OD600 under glucose (Glu, open square), xylose (Xyl, open triangle), cellobiose (Ceb, open circle) and cellulose (Cel, filled circle) and by determining the amount of cellular protein produced on xylan (Xyn, filled triangle) and corn stover (CS, filled square) as described in Methods. The symbols indicate the means of three experiments, and the error bars indicate the standard deviations. (B) Overview of the Clostridium cellulolyticum transcriptome generated by RNA-Seq. Data were normalized by the number of CDS for each function encoded within the entire genome. A ratio of 1 represents transcription of functional class on par with its genome content. A ratio of more than one represents a transcriptionally enriched class, and less than one, depleted. [file 1754-6834-6-73-S2.pdf]

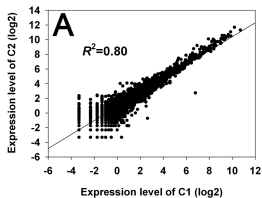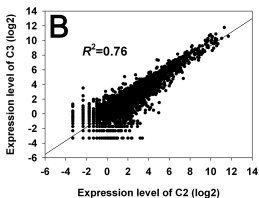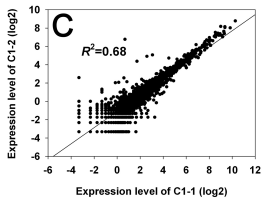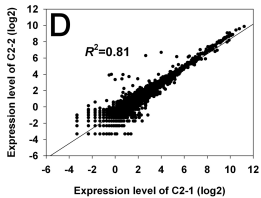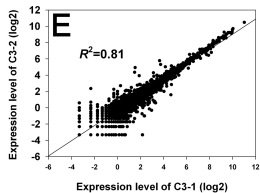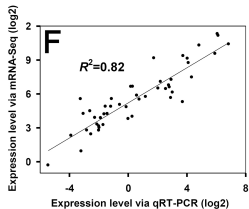

Supplement: Additional file 9: Figure S3 — Validation of mRNA-Seq based transcript quantification for genome-wide expression profiling. (A and B) Biological replicates. In the correlation plots, each point indicates the TA of an individual CDS in two biological replicates for cellulose sample. (C, D, and E) Technical replicates. In the correlation plots, each point indicates the TA of an individual CDS in pairwise technical replicates for cellulose sample. (F) Real-time quantitative RT-PCR (qPCR) validation of mRNA-Seq based transcript quantification. The induction levels were compared among 24 genes in C. cellulolyticum. All genes were randomly selected. The comparison was plotted on log2R, which was determined by qRT-PCR (x axis) and RNA-Seq (y axis). [file 1754-6834-6-73-S9.pdf]
