# Supplementary material for: Structure and regulation of the cellulose degradome in Clostridium cellulolyticum
Source: Biotechnol Biofuels. 2013 May 8;6:73. doi: 10.1186/1754-6834-6-73 (PMC3656788; doi:10.1186/1754-6834-6-73)
Supplement: Additional file 6: Figure S2 — Phylogeny and putative operator motifs of CcpA-like regulators in C. cellulolyticum. (A) Phylogenetic (Neighbor-joining) tree of the HTH DNA-binding domains of Ccel_1005, Ccel_1438, Ccel_2999, Ccel_3000 and Ccel_3464, and their homologues in other Gram-positive bacteria (Methods). Bootstrap values are indicated. (B) Putative operator motifs of the Ccel LacI-family regulators. Upstream regions of the genes flanking the query and subject genes were inspected for sharing similarity to the cre consensus sequence and used to create the Ccel_1005, Ccel_1438, Ccel_2999, Ccel_3000 and Ccel_3464 specific operator motifs. [file 1754-6834-6-73-S6.pdf]

A

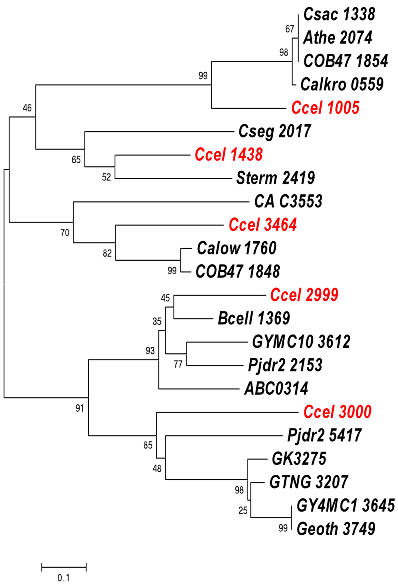

B

	Organism	Gene ID	Putative *cre* site	Consensus logo
Ccel_1005 (CcpA)	*Caldicellulosiruptor obsidiansis*	COB47_1854	AGTAAAACGTTTAGAT	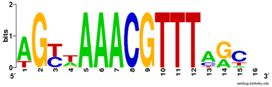
	Caldicellulosiruptor bescii	Athe_2074	TGTAAAACGTTTAGCA	
		Athe_2075	AGCTAAACGTTTCACA	
	Caldicellulosiruptor saccharolyticus	Csac_1338	TGTGAAACGTTTAGCA	
		Csac_1339	AGCTAAACGTTTCACA	
	Caldicellulosiruptor kronotskyensis	Calkro_0559	AGTAAAACGTTTAGTT	
		Calkro_0560	AGCTAAACGTTTAATT	
	Clostridium cellulolyticum	Ccel_1005	AGTTAAACGTTTTGCG	
		Ccel_1006	AGCTAAACGTTTAGCC	
Ccel_1438 (LfpC1)	*Caulobacter segnis*	Cseg_2017	ATGGTAACGTTACCGT	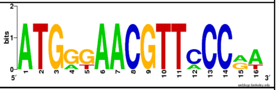
	Clostridium cellulolyticum	Ccel_1438	ATGGGAACGTTCCCAA	
		Ccel_1439	ATGGGAACGTTCCCAA	
	Sebaldella termitidis	Sterm_2419	ATGAGAACGTTCCCGA	
Ccel_2999 (LfpC2)	*Paenibacillus sp. JDR-2*	Pjdr2_5417	TTTGTAACGTTACAAT	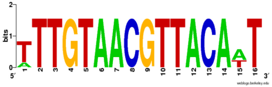
	Geobacillus thermodenitrificans	GTNG_3207	TTTGTAACGTTACAAT	
	Geobacillus kaustophilus	GK_3275	ATTGTAACGTTACAAT	
	Geobacillus sp. Y4.1MC1	GY4MC1_3645	TTTGTAACGTTACATT	
	Geobacillus thermoglucosidasius	Geoth_3749	TTTGTAACGTTACATT	
Ccel_3000 (LfpC3)	*Clostridium cellulolyticum*	Ccel_2997	AGCTTAACGTTAATCA	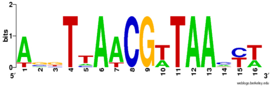
	Bacillus cellulosilyticus	Bcell_1369	TAGTCAACGTTAACTT	
	Bacillus clausii	ABC0314	ACTTTATCGTTAAACT	
		ABC0315	ACTTAAACGTTAACTT	
	Paenibacillus sp. JDR-2	Pjdr2_2153	AGGTTAACGATAAACA	
	Geobacillus sp. Y412MC10	GYMC10_3612	AGGTTAACGATAACTT	
Ccel_3464 (LfpC4)	*Clostridium acetobutylicum*	CA_C3553	AGTATAACGTTGTACC	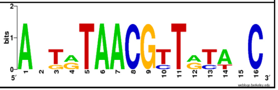
	Clostridium cellulolyticum	Ccel_3464/3463	ACGATAACGCTATTTC	
	Caldicellulosiruptor owensensis	Calow_1760	ATTGTAACGTTGTAGC	
	Caldicellulosiruptor obsidiansis	COB47_1848	ATTGTAACGTTACAGC	
