# Supplementary material for: Structure and regulation of the cellulose degradome in Clostridium cellulolyticum
Source: Biotechnol Biofuels. 2013 May 8;6:73. doi: 10.1186/1754-6834-6-73 (PMC3656788; doi:10.1186/1754-6834-6-73)
Supplement: Additional file 8: Table S6 — Label-free quantitation of proteins in cell-free supernatant of Clostridium cellulolyticum based on the normalized spectra counts of LC-MS/MS. [file 1754-6834-6-73-S8.doc]

**Table S6. Label-free quantification of proteins in cell-free supernatant based on the normalized spectra counts of LC-MS/MS**

| **Locus** | **Annotation** | **Cellobiose** | | | **Glucose** | | | **Fold Change^b^** |
| --- | --- | --- | --- | --- | --- | --- | --- | --- |
|  |  | **1** | **2** | **3^a^** | **1** | **2** | **3^a^** |  |
| Ccel_0231 | glycoside hydrolase family 9 | 0 | 0 | 0 | 1 | 2 | 1 | 2.0 |
| Ccel_0728 | cellulosome scaffoldin protein CipC | 9 | 9 | 3 | 31 | 26 | 22 | 1.9 |
| Ccel_0729 | glycoside hydrolase family 48 | 128 | 147 | 156 | 128 | 111 | 70 | -0.5 |
| Ccel_0731 | glycoside hydrolase family 9 | 0 | 0 | 0 | 3 | 13 | 0 | 4.0 |
| Ccel_0732 | glycoside hydrolase family 9 | 66 | 66 | 53 | 103 | 117 | 92 | 0.8 |
| Ccel_0750 | glycoside hydrolase family 11 | 0 | 0 | 0 | 17 | 22 | 16 | 5.8 |
| Ccel_0753 | glycoside hydrolase family 9 | 0 | 0 | 0 | 3 | 3 | 5 | 3.5 |
| Ccel_0755 | glycoside hydrolase family 9 | 4 | 4 | 0 | 39 | 50 | 48 | 4.1 |
| Ccel_0931 | glycoside hydrolase family 10 | 0 | 0 | 0 | 4 | 2 | 3 | 3.2 |
| Ccel_1099 | glycoside hydrolase family 5 | 0 | 0 | 0 | 2 | 1 | 0 | 1.6 |
| Ccel_1207 | cellulosome protein dockerin type I | 0 | 0 | 0 | 4 | 5 | 3 | 3.6 |
| Ccel_1648 | glycoside hydrolase family 9 | 0 | 0 | 0 | 5 | 4 | 4 | 3.7 |
| Ccel_1809 | cellulosome protein dockerin type I | 5 | 7 | 3 | 2 | 3 | 1 | -1.3 |
| Ccel_1987 | solute-binding component of ABC transporter | 3 | 4 | 2 | 74 | 77 | 75 | 4.7 |
| Ccel_2112 | solute-binding component of ABC transporter | 307 | 334 | 341 | 1 | 3 | 4 | -6.9 |
| Ccel_2222 | fructose-1,6-bisphosphate aldolase | 12 | 8 | 8 | 1 | 2 | 2 | -2.5 |
| Ccel_0212 | phosphoenolpyruvate carboxykinase (GTP) | 27 | 23 | 20 | 12 | 13 | 0 | -1.5 |
| Ccel_0429 | PKD domain containing protein | 31 | 20 | 19 | 11 | 6 | 0 | -2.0 |
| Ccel_1940 | Nucleotidyl transferase | 0 | 0 | 0 | 4 | 4 | 3 | 3.5 |
| Ccel_3435 | ketol-acid reductoisomerase | 0 | 0 | 0 | 0 | 1 | 0 | 0 |
| Ccel_0623 | hypothetical protein | 0 | 0 | 0 | 5 | 4 | 6 | 3.9 |
| Ccel_0835 | hypothetical protein | 16 | 19 | 16 | 0 | 0 | 0 | -5.7 |
| Ccel_1426 | hypothetical protein | 11 | 10 | 9 | 0 | 0 | 0 | -4.9 |
| Ccel_2789 | hypothetical protein | 7 | 5 | 0 | 0 | 0 | 0 | -3.6 |
| Ccel_3398 | hypothetical protein | 124 | 123 | 125 | 41 | 48 | 35 | -1.6 |

^a^Three technical replicates.

^b^ The fold change was calculated by log_2_ (Sum of counts under glucose/Sum of counts under cellobiose). If the sum of spectral counts was “0”, it was set to “1” which was the minimum of spectral counts for the calculation of the fold change.
