# Supplementary material for: Structure and regulation of the cellulose degradome in Clostridium cellulolyticum
Source: Biotechnol Biofuels. 2013 May 8;6:73. doi: 10.1186/1754-6834-6-73 (PMC3656788; doi:10.1186/1754-6834-6-73)
Supplement: Additional file 10: Table S7 — A complete list of PCR primer sets used in this study. [file 1754-6834-6-73-S10.doc]

**Table S7. A complete list of PCR primer sets used in this study**

| **Primers** | **Sequences (5'-3')a** | **Description** |
| --- | --- | --- |
| For Real-time quantitative RT-PCR (qPCR) | |  |
| Ccel_0270_F | TTGCACAGTTCGGTTCTGAC | Intragenic region of Ccel_0270, qRT-PCR |
| Ccel_0270_R | CACAAGCTCCCGGTTGAACT |  |
| Ccel_0271_F | ATGAGCCGTCAGTAACAGAA | Intragenic region of Ccel_0271, qRT-PCR |
| Ccel_0271_R | AGTTATAGTCGCCTGTCTTG |  |
| Ccel_0312_F | GCCAGTTGGTGAGATCCCCT | Intragenic region of Ccel_0312, qRT-PCR |
| Ccel_0312_R | ACCGTACCCTAATGCTCTGA |  |
| Ccel_0297_F | ACAAGGGACCGTTTAACCTC | Intragenic region of Ccel_0297, qRT-PCR |
| Ccel_0297_R | GCCCGAAATAGTCTCCTCGT |  |
| Ccel_0298_F | AAGTCAAACCACGGAGACTG | Intragenic region of Ccel_0298, qRT-PCR |
| Ccel_0298_R | CCATATCAGCCAGACTGCTT |  |
| Ccel_0445_F | ACAGTATCTATCCCTGAAGC | Intragenic region of Ccel_0445, qRT-PCR |
| Ccel_0445_R | TCGCCCTCTTTTTTAGCCTG |  |
| Ccel_0446_F | CTCTTTCTGTAACAGCCTT | Intragenic region of Ccel_0446, qRT-PCR |
| Ccel_0446_R | AGAGGGGTTTACATACAAGT |  |
| Ccel_0597_F | TCAGGCAAGGTTGTAAACAT | Intragenic region of Ccel_0597, qRT-PCR |
| Ccel_0597_R | CCGTCAAGTGCATATACAGA |  |
| Ccel_0598_F | CTTTGAGATGATTTTGGTGG | Intragenic region of Ccel_0598, qRT-PCR |
| Ccel_0598_R | CAATAAGCAGAAATGCAACA |  |
| Ccel_0728_F | ACGACTGGAGTAATTTCGAT | Intragenic region of Ccel_0728 (*cipC*), qRT-PCR |
| Ccel_0728_R | AGTATCTGCGAAAGATCCTG |  |
| Ccel_0729_F | ACTATAGCGATTCAAAGGGT | Intragenic region of Ccel_0729 (*celF*), qRT-PCR |
| Ccel_0729_R | TAATGCAGCAACCATTGTCT |  |
| Ccel_0730_F | TATGTCCTGGCGTATTGACT | Intragenic region of Ccel_0730 (*celC*), qRT-PCR |
| Ccel_0730_R | ATGTACCCGGTTCTACCATT |  |
| Ccel_0731_F | CACGGTTCATGGACAGATCA | Intragenic region of Ccel_0731, qRT-PCR |
| Ccel_0731_R | GGATCTCCGCCAGAATGCTT |  |
| Ccel_0732_F | TTGCAGCCTCCAAGTACAGC | Intragenic region of Ccel_0732, qRT-PCR |
| Ccel_0732_R | TTGTCTCCGTATGCTCCACC |  |
| Ccel_0885_F | TGCTTCCGACGCACTTGCTA | Intragenic region of Ccel_0885, qRT-PCR |
| Ccel_0885_R | CGGCAGCCAGTGATTCACCC |  |
| Ccel_1060_F | GACTGCATAGGCGGAAGCGT | Intragenic region of Ccel_1060, qRT-PCR |
| Ccel_1060_R | TTTGGCGTTGTTGAACTGGT |  |
| Ccel_1223_F | ATCTGCTGAAATAGGCCGTA | Intragenic region of Ccel_1223, qRT-PCR |
| Ccel_1223_R | AAGCATCAATAGCTCCGAAG |  |
| Ccel_1608_F | ATGGGGATACCACAAGCAGA | Intragenic region of Ccel_1608, qRT-PCR |
| Ccel_1608_R | TGAGCAGTATCTCCGTCCGT |  |
| Ccel_1986_F | TTCCCGAAACGTACACCCCA | Intragenic region of Ccel_1986, qRT-PCR |
| Ccel_1986_R | CCGTAAGCCTTTCCGAACAC |  |
| Ccel_1987_F | CGCTATTGACTGTTCAGCAC | Intragenic region of Ccel_1987, qRT-PCR |
| Ccel_1987_R | TGAATGGTCCTTTGCCATCT |  |
| Ccel_2065_F | TGACGTTGTTCCCAGACAGT | Intragenic region of Ccel_2065, qRT-PCR |
| Ccel_2065_R | ATTCCCCTGCCGAAAAGCCT |  |
| Ccel_2066_F | ATTTCACATATGTGGCTGTT | Intragenic region of Ccel_2066, qRT-PCR |
| Ccel_2066_R | ACCAAAGGCAAAAATAGCTG |  |
| Ccel_2109_F | TAATCCTGCCGGAGTTCAGA | ntragenic region of Ccel_2109 (*cdpA*), qRT-PCR |
| Ccel_2109_R | AACCCATACCCCTTCCGATA |  |
| Ccel_2111_F | ATCAGGAACAATCAACGCAA | Intragenic region of Ccel_2111 (*cuaB*), qRT-PCR |
| Ccel_2111_R | CTCCATCAACACGTGCAGCC |  |
| Ccel_2112_F | AGGCTGGCGTTATCAACAAA | Intragenic region of Ccel_2112 (*cuaA*), qRT-PCR |
| Ccel_2112_R | TGACAATGCAGTACCACCCC |  |
| Ccel_2485_F | CCAGAAGCCCGGTGAAACCA | Intragenic region of Ccel_2485, qRT-PCR |
| Ccel_2485_R | CAAGAACTGTTCCGGAGCCT |  |
| Ccel_R0018-F | ACCCTGGAGCTGCATCTGAA | Intragenic region of Ccel_R0018 (16S rRNA), qRT-PCR |
| Ccel_R0018-R | CATCGTTTACAGCGTGGACT |  |
